# Bio-surveillance of environmental pollutants in the population of Kinshasa, Democratic Republic of Congo (DRC): a small pilot study

**DOI:** 10.1186/s13690-021-00717-x

**Published:** 2021-11-15

**Authors:** Trésor Bayebila Menanzambi, Patrice Dufour, Catherine Pirard, Jean Nsangu, Jean-Pierre Mufusama, Jérémie Mbinze Kindenge, Roland Marini Djang’eing’a, Corinne Charlier

**Affiliations:** 1 Faculty of Pharmaceutical Sciences, University of Kinshasa, Kinshasa, Democratic Republic of the Congo; 2 Laboratory of Clinical, Forensic and Environmental Toxicology, University of Liege (ULiege), CHU (B35), 4000 Liege, Belgium; 3 Center for Interdisciplinary Research on Medicines (C.I.R.M), University of Liege (ULiege), CHU (B35), 4000 Liege, Belgium

**Keywords:** Bio-surveillance, Small pilot study, Environmental pollutants, Urine, Serum, Whole blood, Kinshasa

## Abstract

**Background:**

Environmental pollutants are known to be ubiquitous and may present toxic effects (endocrine-disruption properties, carcinogenicity …) and represent a real threat to human health. The aim of the present pilot study was to assess the content of environmental pollutants (inorganic, persistent, and non-persistent pollutants) in biological samples (urine, serum, and whole blood), collected from volunteers in Kinshasa, capital of Democratic Republic of Congo, in order to identify pollutants of interest and to design a protocol for a larger scale study.

**Methods:**

From randomly selected 15 volunteers living in Kinshasa, aged from 25 to 66 years, (mean age = 43.4 years), including 10 men and 5 women, urine, whole blood, and serum samples were used in this study to estimate the contents in these environmental pollutants, using inductively coupled plasma mass spectrometry, gas chromatography coupled to mass spectrometry, and liquid chromatography coupled to mass spectrometry.

**Results:**

When compared to data nationally and internationally available, the preliminary outcomes of this study indicated a very high level of exposure to environmental pollutants in the population of Kinshasa, especially for heavy metals, parabens and triclosan. To a lesser extent, contamination measured for glyphosate, phthalates, organochlorine pesticides, pyrethroids and dialkylphosphate pesticides was also significant. In contrast, the investigated population of Kinshasa was found to be weakly exposed to other persistent organic pollutants like polychlorinated biphenyls, brominated flame retardants, phenolic organohalogens, and perfluoroalkyl substances.

**Conclusion:**

Although the biologic fluids were collected from a limited number of volunteers (*n* = 15), the results of the present report clearly indicate that the population of Kinshasa is not spared by the investigated environmental pollutants. Moreover, this study gives us important information to design a larger scale study protocol.

**Supplementary Information:**

The online version contains supplementary material available at 10.1186/s13690-021-00717-x.

## Background

Environmental protection is a key to the sustainable development. For decades, the environment has indeed been threatened by different human activities due to industrialization, including progress in agriculture, growing use of plastic materials, fire management products, pharmaceuticals, and cosmetics [1]. Since decades, the correlation between the increase in chemical production and that of the chronic disease prevalence has suggested that some chemicals may be responsible of endocrine disruptions, carcinogenicity, or other toxic effects [2–4]. As these compounds are ubiquitous and can operate at low concentrations, their release in the environment poses a potential threat to human health [5, 6]. The actual systemic exposure of an individual to environmental pollutants can be evaluated by the quantification of these compounds or their metabolites in biological fluids [7].

Although being a controversy topic, many bio-surveillance studies have reported harmful effects of environmental pollutants in humans. Among compounds presumed to have health-threatening properties, on one hand, persistent organic pollutants (POPs) are organic compounds with remarkable resistance to degradation into environment and among which there are organochlorine pesticides (OCP), polychlorinated biphenyls (PCB), brominated flame retardants (BFR), phenolic organo-halogens (POH), and perfluoroalkyl substances (PFAS). Among the health concerns associated to these compounds we could mention diseases affecting the central nervous system, metabolic diseases, and birth weight alteration [8]. On the other hand, inorganic pollutants (IP), especially arsenic, cadmium, lead, cobalt, tin, etc., are mineral compounds and many of them are likely used in several industrial activities. Some of them are alleged of being neurotoxic and of having damaging effects to noble organs like kidney, liver, heart, etc. [9]. Furthermore, non-persistent organic pollutants (nPOPs) like pyrethroids, alkyl-phosphates, bisphenols, triclosan, phthalates, parabens, glyphosate, and benzophenone, are organic compounds with fast degradation into environment but industrially produced in large amounts and are suspected to be responsible of several dysfunctions of the hormonal systems (reproductive or thyroid system), central nervous system, and in the occurrence of metabolic and chronic diseases [10].

With an area of 9965 km^2^ and an estimated population of 14.3 Million of inhabitants in 2020, Kinshasa is the capital, the most populated and the biggest city of the Democratic Republic of the Congo (DRC). Besides an important demographic increase, there is also an increase in morbidity and mortality rates due to chronic and metabolic diseases [11–13]. Moreover, Tuakuila J. et al. 2015 [14] reported a lack of data on bio-surveillance of environmental pollutants in the population of Sub-Saharan African countries in general and particularly in DRC. This lack of data poses a big limitation in the surveillance of exponentially growing pathologies and highlights that very few resources are dedicated to the biomonitoring of environmental pollutants in Sub-Saharan Africans populations. Consequently, before performing large scale investigations, pilot studies are required to identify compounds that contaminate individuals at high levels. Identifying such pollutants would help to coherently attribute limited resources to the study of compounds potentially associated with higher health risks which subsequently could prompt local stakeholders to implement targeted and effective environmental policies.

In the present study, we assessed the content of environmental pollutants (inorganic, persistent, and non-persistent pollutants) in biological samples (urine, serum, and whole blood), collected from 15 volunteers aging from 25 to 66 years (mean age = 43.4 years), belonging to various business sectors, and living in Kinshasa. The aim of the investigation was to estimate roughly the exposure level of the population of Kinshasa to environmental pollutants and to identify chemicals that are present at high levels. This information will help us to write a study protocol for a future largescale study targeting compounds associated with potential high health impact on Kinshasa residents. To the best of our knowledge, this is the first study reporting on bio-surveillance data of organic persistent and non-persistent pollutants in the population of Kinshasa as well as in that of DRC.

## Methods

### Sample collection

Biological fluids were collected from 15 volunteers recruited in the population of Kinshasa with ages ranging from 25 to 66 years, the mean and the median age were 43.4 and 47.0 years respectively (SD = 14.3 years), including 10 men and 5 women. For this small pilot bio-surveillance study, with the aim to conduct the first exploration of pollutant contamination in the general population of Kinshasa and covering various exposition profiles, volunteers were selected among business sectors, including market gardeners, pesticide vendors, plastic manufacturers, aluminium utensil makers, mechanics, traders, students, lawyers, painters, drivers, teachers, polices, students, fitters, and sanitation technicians. In each sector, a volunteer was randomly chosen throughout the city. Prior to be enrolled, presumed volunteers belonging to various business sectors were directly informed about the study merits by the principal investigator in their workplace. One individual per sector was randomly selected among people who agree to participate to the study. This volunteer was invited to fulfil the consent form and was submitted to a questionnaire to record his/her age, business activity, commonly handled products, and duration of exposure. All fifteen volunteers filled out the questionnaire with the help of the principal investigator. Volunteers had to be over 18 years old, have worked full time for at least 1 year in the same workplace and individuals who were out of the city for at least 6 months for the last decade were excluded from the recruitment.

Early in the morning, after breakfast, each volunteer was requested to give about 10 mL of whole blood, kept in a plastic tube (without gel but with heparin), 10 mL of whole blood to prepare the serum, kept in a plastic tube (without gel and without heparin), and 50 mL of urine, kept in a polypropylene vial, all together 45 samples for analysis. These samples were collected between March and April 2019 and placed immediately in a dry ice enclosure, while ensuring that tubes with whole blood were not in direct contact with the ice, to avoid the risk of haemolysis and facilitating their transport, for proper storage, to the Clinical Biology Laboratory of the Faculty of Pharmaceutical Sciences at the University of Kinshasa. Tubes with whole blood for the preparation of the serum samples were centrifuged for 5 min at 3000 rpm and kept, together with urine samples, in a freezer at − 20 °C, while tubes of heparinized whole blood were stored in a fridge at 4 °C. Toxicological analyses were carried out in the Laboratory of Clinical, Forensic and Environmental Toxicology, at the University of Liege, in Belgium. For a proper transport to Belgium, all samples were stored in a hermetically sealed enclosure with dry ice, while ensuring that the whole blood tubes were not in direct contact with the ice. The current study was approved by the national health ethics committee in the Congo under the series number of 159/CNES/BN/PMMF/2020.

### Analytical procedures

#### Analysis of metals and metalloids in urine

The inorganic compounds (namely, As, Bi, Cd, Co, Cr, Cu, Mn, Mo, Ni, Sb, Se, Sn, Tl, V and Zn) were analysed using ICP-MS. Briefly, internal standard (containing Rh, Sc and Ge) was added, at the same time, to the sample, to the quality control sample, and to the standard calibration sample. This mixture was then diluted with an aqueous solution of nitric acid 0.5% before being injected into ICP-MS. For detailed analytical methodology, see supplementary information.

#### Analysis of glyphosate in urine

Urinary content in glyphosate was investigated following the procedure extensively described in the supplementary information. Briefly, urinary glyphosate in sample, quality control sample, and standard calibration sample was derivatized with fluorenylmethoxycarbonyl chloride (FMOC). A first liquid-liquid extraction was then performed to eliminate residual FMOC and nonpolar compounds. A second liquid-liquid extraction was performed after acidification to extract the analyte. After evaporation and reconstitution in vial, the sample was analysed by LC-MS/MS.

#### Analysis of pyrethroids and organophosphate chlorpyrifos metabolites in urine

Five pyrethroids metabolites (namely, cis- and trans-3-(2,2-dichlorovinyl)-2.2-dimethylcyclopropane carboxylic acid (c- and t-DCCA), 3-phenoxybenzoic acid (3-PBA), 4-fluoro-3-phenoxybenzoic acid (4F-3-PBA) and 3-(2,2-dibromovinyl)-2.2-dimethylcyclopropane carboxylic acid (DBCA)) and one chlorpyrifos metabolite (namely 3,5,6-trichloro-2-pyridinol (TCPY)) were analyzed according to the methodology detailed in Pirard et al. 2020 [15]. Briefly, urinary sample, quality control sample, and standard calibration sample were extracted with diethyl ether. The organic layer was evaporated to dryness and the residue was derivatized with N-tert-butyldimethylsilyl-N-methyltrifluoroacetamide (MTBSTFA). The derivatized extract was then analysed by GC-MS/MS.

#### Analysis of alkylphosphates in urine

Five dialkylphosphates (DAPs) (nonspecific metabolites of organophosphate pesticides) (namely dimethylthiophosphate (DMTP), dimethyldithiophosphate (DMDTP), diethylphosphate (DEP), diethylthiophosphate (DETP) and diethyldithiophosphate (DEDTP)) were quantified in urine samples according to the methodology described in Pirard et al., 2020 [15]. In summary, urine sample, quality control sample, and standard calibration sample were extracted on solid phase extraction (SPE) cartridge. The eluate was evaporated to dryness and then derivatized with chloroiodopropane. The derivatized extract was then analysed by GC-MS/MS.

#### Analysis of phthalate metabolites, parabens, and benzophenone-3 in urine

The urinary concentrations of 7 phthalate metabolites (namely, monoethyl phthalate (MEP), mono-iso-butyl phthalate (MiBP), mono-n-butyl phthalate (MnBP), monobenzyl phthalate (MBzP), mono-2-ethylhexyl phthalate (MEHP), mono-2-ethyl-5-hydroxyhexyl phthalate (5-OH-MEHP) and mono-2-ethyl-5-oxohexyl phthalate (5-oxo-MEHP)), 4 parabens (namely, methylparaben (MeP), ethylparaben (EP), n-propylparaben (PrP) and n-butylparaben (BP)) and benzophenone-3 were determined according to the methodology developed by Dewalque et al. 2014 [16]. Briefly, urine sample, quality control sample, and standard calibration sample were submitted to an enzymatic hydrolysis, then an extraction was performed using SPE cartridge and finally the extract was analysed on a LC-MS/MS apparatus.

#### Analysis of triclosan and bisphenols in urine

The levels of triclosan and 7 bisphenols (BP) (namely, BPA, BPAF, BPF, BPZ, BPAP, BPP and BPS) in urine samples were measured by using the methodology detailed in the supplementary materials. In summary, the sample, quality control sample, and standard calibration sample were submitted to an enzymatic hydrolysis followed by an extraction on a SPE cartridge. This first extraction was followed by a liquid-liquid extraction and then by a derivatization. The derivatized extract was then analysed by a GC-MS/MS [17].

#### Analysis of lead in whole blood

Lead was quantified in whole blood. Samples, quality control samples, and standard calibration samples were mixed with internal standard and diluted with a mixture of nitric acid (0.5%), n-butanol (0.2%) and triton (0.1%) in water. The lead content was determined by using an ICP-MS. The procedure has been detailed in supplementary information.

#### Analysis of polychlorobiphényls (PCBs) and organochlorine pesticides in serum

Fifteen organochlorine pesticides or metabolites, (namely alpha-, beta-and gamma-HCH (α-, β- and γ-HCH), hexachlorobenzene (HCB), aldrin, dieldrin, endrin, trans-chlordane, oxychlordane, trans-heptachlor epoxide, *cis*- and *trans*-nonachlor, 2,4′- and 4,4′-dichlorodiphenyl-dichloroethylene (DDE), beta-endosulfan) and 3 PCBs (− 138, − 153, and − 180) were quantified in serum. The analytical procedure was extensively detailed in Pirard et al. 2018 [1]. Briefly, sample, quality control sample, and standard calibration sample were denaturized with acetonitrile and a saturated potassium carbonate solution. The mixture was then extracted twice with hexane-acetone mixture (9/1, v/v). The organic phase was cleaned on a SPE cartridge and then evaporated and reconstituted in nonane. The extract was analysed on a GC-MS/MS apparatus.

#### Analysis of BFRs in serum

The methodology to quantify 8 polybrominated diphenylethers (PBDEs) (namely, BDE-28, − 47, − 99, − 100, − 153, − 154, − 183 and − 209) has been described in Pirard and Charlier, 2018 [18]. In summary, serum sample, quality control sample, and standard calibration sample were denaturized with a glacial acetic acid/water mixture (3/7, v/v) and then extracted twice with a mixture of hexane and acetone (95/5, v/v). The organic phase was then cleaned on a PHREE cartridge, then evaporated and transferred into nonane. The quantification was performed using a GC-MS/MS.

#### Analysis of perfluorinated alkyl subtances (PFAS) in serum

The quantification of the serum content in 7 PFASs (namely, perfluoro-octane sulfonic (PFOS), perfluoroctanoic acid (PFOA), perfluorohexane sulfonate (PFHxS), perfluorononanoic acid (PFNA), perfluorodecanoic acid (PFDA), perfluoroheptanoic acid (PFHpA) and perfluoroundecanoic acid (PFUdA)) was performed according to the methodology described in Dufour et al. 2018 [8]. Briefly, serum sample, quality control sample, and standard calibration sample were denaturized with formic acid/water mixture (1/1, v/v). Then the sample was extracted on a weak anionic exchange SPE cartridge, the eluate was evaporated to dryness and then reconstituted in 80 μL of a mixture of mobile phases. The extract was then analysed using a LC-MS/MS apparatus.

#### Analysis of phenolic organohalogens (POHs) in serum

POHs (namely, pentachlorophenol (PCP), tetrabromobisphenol A (TBBPA), 2,4,6-tribromophenol (2,4,6-TBP), 2,3,6-tribromophenol (2,3,6-TBP), 2,4,5-tribromophenol (2,4,5-TBP), 2,3,4,6-tetrabromophenol (2,3,4,6-TeBP), 6-hydroxy-polybromodiphenylether 47 (6-OH-BDE 47), 5-hydroxy-polybromodiphenylether 47 (5-OH-BDE 47), 5′-hydroxy-polybromodiphenylether 99 (5′–OH–BDE 99), 4-hydroxy-polychlorinated biphenyl 107 (4-OH-CB 107), 3-hydroxy-polychlorinated biphenyl 138 (4-OH-CB 138), 4-hydroxy-polychlorinated biphenyl 146 (4-OH-CB 146), 3-hydroxy-polychlorinated biphenyl 153 (3-OH-CB 153), 4-hydroxy-polychlorinated biphenyl 172 (4-OH-CB 172), 3-hydroxy-polychlorinated biphenyl 180 (3-OH-CB 180) and 4-hydroxy-polychlorinated biphenyl 187 (4-OH-CB 187)) were analysed according to the method described in Dufour et al. 2016 [19]. In summary, the serum sample, quality control sample, and standard calibration sample were denaturized with a mixture of water/formic acid/2-propanol (50/40/10, v/v) and then extracted on a strong anionic exchange SPE cartridge. The eluate is then extracted with hexane; hexane phase was evaporated to dryness and then derivatized with trimethylsilyldiazomethane. The derivatized extract was then analysed using a GC-MS/MS.

#### Analysis of creatinine in urine samples

Adjustment to creatinine was used to normalize pollutant contents in urine samples. In this study, urinary creatinine was evaluated on an ARCHITECT Ci 4100 automate from ABBOTT (Illinois, USA), using an immunoassay.

#### Quality assurance and statistical analysis

To ensure the results quality, all analyses were covered by internal or external quality control sample and for each analysis, a specific internal standard was used as a recovery indicator and a correction factor. A specific calibration curve was applied for each analysis. All statistical analyses were performed using R programming software (version 3.6.3., CRAN) and Microsoft Excel 2013 (Microsoft, Redmond, WA). For analyses with results lower than the limit of quantification (LOQ), a correction was made by multiplying the LOQ by the detection frequency (DF), in order to valorise the investigation outcomes.

#### Comparison with data reported in the literature

In order to identify chemicals for which high concentration are measured in Kinshasan population, we compared our results with those reported in other studies performed on general populations originated from other countries around the world. First, we performed systematic research in Pubmed to identify the studies reporting pollutant contamination levels in Sub-Saharian Africans populations (we also included RDC). Secondly, because biomonitoring investigations are relatively numerous in Belgium, because contamination measured in Belgian population can be considered as roughly representative of the situation in Western Europe and because the Laboratory of Toxicology of the University Hospital of Liege is particularly involved in the biomonitoring of the Belgian population, for each family of pollutants we compared our data with those reported in studies performed on general Belgian population. Finally, we searched in Pubmed, articles reporting pollutant contamination measured in general populations around the world, the aim was not to perform a systematic review of the literature so we reported only few examples for each compounds family and we were attentive to exclude studies assessing populations with identified significant source of contamination.

## Results

Tables 1 and 2 gather results for the most significant and most representative compounds for each family of pollutants. The results for the entire set of chemicals are reported in supplementary information.

**Table 1:** LOQ of methods (pg/mL) and DF (%) of environmental pollutants in Kinshasa

	matrix	pollutant	LOQ	DF		matrix	pollutant	LOQ	DF
***Inorganics Pollutants***					***nPOPs***				
	Urine	Vanadium	180	73		*Urine*	*Parabens*		
		Chrome	230	100			MeP	790	100
		Manganese	890	20			EP	300	67
		Cobalt	130	100			PrP	360	100
		Nickel	1630	100			*Phthalates*		
		Cadmium	120	93			MEP	940	100
		Tin	730	47			MEHP	620	100
		Antimony	140	47			MnBP	990	100
		Thalium	90	100			MiBP	1230	100
		Bismuth	120	100			MBzP	610	87
		Copper	1600	100			*Benzophenone*		
		Zinc	15,000	100			BP3	670	100
		Arsenic	140	100			*Pyrethroid pesticides*		
		Selenium	7900	100			c-DCCA	150	87
		Molybdene	5000	100			t-DCCA	150	93
	Blood	Lead	500	100			TCPY	80	100
***POPs***							DBCA	300	87
	*Serum*	*Organochlorine pesticides*					FPBA	110	7
		4,4′-DDE	400	87			3-PBA	90	100
		HCB	80	7			*Triclosan*		
		β-HCH	50	0			TCS	200	100
		*Polychlorinated biphenyls*					*Bisphenols*		
		PCB 153	70	60			BPF	70	67
		PCB 138	150	7			BPA	290	100
		PCB 180	50	53			BPZ	60	0
		*Perfluoroalkyl substances*					BPS	90	80
		PFOA	250	100			*Organophosphate pesticide*		
		PFOS	500	47			DEP	500	53
		PFHxS	150	60			DETP	500	47
		PFNA	100	60			*Glyphosate*		
		*Brominated flame retardants*					Glyphosate	80	93
		PBDE 47	3.7	27					
		PBDE 153	4.2	27					
		*Phenolic organohalogens*							
		PCP	44.6	14					
		2,4,6 TBP	49.6	0					
		4-OH CB 146	2.2	29					
		4-OH CB 187	2	57					
		6-OH BDE 47	2.5	0					

*LOQ* Limit of Quantification,

*DF* Detection Frequency

**Table 2:** Mean, geometric mean, median and range concentrations in urine [μg/L (μg/g creatinine)] and at both blood and serum in μg/L

	Pollutant	Mean	Geometric mean	Median	Minimum	Maximum
***Urinary***	Cobalt	1.0 (0.31)	0.57 (0.24)	0.43 (0.21)	0.16 (0.089)	6.11 (0.87)
	Cadmium	1.15 (0.33)	0.66 (0.28)	0.61 (0.26)	‹LOQ (‹LOQ)	6.12 (0.87)
	Arsenic	81.74 (32.33)	69.73 (29.79)	70.91 (30.86)	32.12 (11.97)	215.32 (57.28)
	Glyphosate	0.22 (0.095)	0.19 (0.083)	0.23 (0.098)	0.09 (0.05)	0.40 (0.18)
	MeP	699.98 (335.48)	216.12 (92.32)	445.39 (121.96)	15.06 (9.94)	4467.5 (1386.5)
	EP	11.94 (2.96)	0.73 (0.31)	0.47 (0.25)	‹LOQ (‹LOQ)	86.5 (26.83)
	PrP	290.62 (157.53)	38.96 (16.64)	31.35 (5.35)	0.97 (0.65)	2509.15 (778.75)
	MEP	309.94 (96.09)	113.09 (48.31)	108.56 (41.96)	13.96 (9.45)	2366.9 (734.6)
	MEHP	16.47 (6.26)	8.53 (3.64)	9.03 (3.25)	0.97 (0.84)	62.45 (15.75)
	MnBP	229.67 (92.86)	176.42 (75.36)	145.19 (60.72)	32.05 (31.79)	638.11 (292.3)
	MiBP	31.39 (13.42)	25.26 (10.79)	26.52 (9.32)	7.43 (4.1)	72.37 (27.97)
	BP3	6.76 (2.59)	4.97 (2.12)	5.00 (1.93)	0.76 (0.65)	23.31 (6.67)
	c-DCCA	0.81 (0.28)	0.41 (0.18)	0.47 (0.23)	‹LOQ (‹LOQ)	3.9 (0.69)
	t-DCCA	1.34 (0.47)	0.72 (0.31)	0.59 (0.31)	‹LOQ (‹LOQ)	0.17 (0.12)
	TCPY	37.68 (13.52)	9.19 (3.92)	4.43 (2.19)	0.40 (0.44)	123.36 (54.6)
	3-PBA	16.88 (4.84)	3.36 (1.44)	2.25 (1.22)	0.29 (0.15)	173.20 (48.50)
	TCS	90.44 (44.45)	40.88 (17.46)	40.13 (17.76)	4.48 (0.64)	277.82 (184.70)
	BPA	1.96 (0.88)	1.62 (0.69)	1.36 (0.76)	0.52 (0.38)	5.40 (2.08)
	DEP	4.30 (1.24)	0.30 (0.13)	0.87 (0.19)	‹LOQ (‹LOQ)	32.70 (9.18)
	DETP	1.95 (0.63)	0.76 (0.33)	0.35 (0.24)	‹LOQ (‹LOQ)	9.23 (2.34)
***Blood***	Lead	62.96	53.69	53.57	22.34	156.96
***Serum***	4,4’DDE	3.02	1.69	1.46	‹LOQ	9.20
	PCB 153	0.09	0.08	0.08	‹LOQ	0.20
	PFOA	0.49	0.47	0.48	0.25	0.85
	PFOS	0.58	‹LOQ	0.50	‹LOQ	1.54
	PCP	‹LOQ	‹LOQ	‹LOQ	‹LOQ	102.4^a^
	4-OH CB187	7.48^a^	‹LOQ	2.44^a^	‹LOQ	20.4^a^
	PBDE47	‹LOQ	‹LOQ	‹LOQ	‹LOQ	15.06^a^
	PBDE 153	‹LOQ	‹LOQ	‹LOQ	‹LOQ	13.45^a^

^a^concentration in pg/mL

*LOQ* Limit of Quantification

Table 1 shows the qualification limits of the used methods for each pollutant as well as their detection frequencies, respectively in pg/mL and in percentage (%).

Table 2 presents arithmetic and geometric means, medians, minimum and maximum contents in urine, whole blood, and serum samples of some pollutants or their corresponding biomarkers. Being robust at extreme values, the median concentrations were considered as reference points.

Results obtained from urine samples analyses were presented in μg/L, and in μg/g of creatinine in parenthesis. Many bio-surveillance studies have suggested that only considering the expression of concentration adjusted by creatinine could lead to a bias when comparing different populations, pollutants contents in urine samples in comparative studies were only reported in μg/L [20, 21]. Analyses of serum and whole blood samples are expressed in μg/L or in pg/mL (different unities were used to facilitate study comparison).

**Fig. 1:**
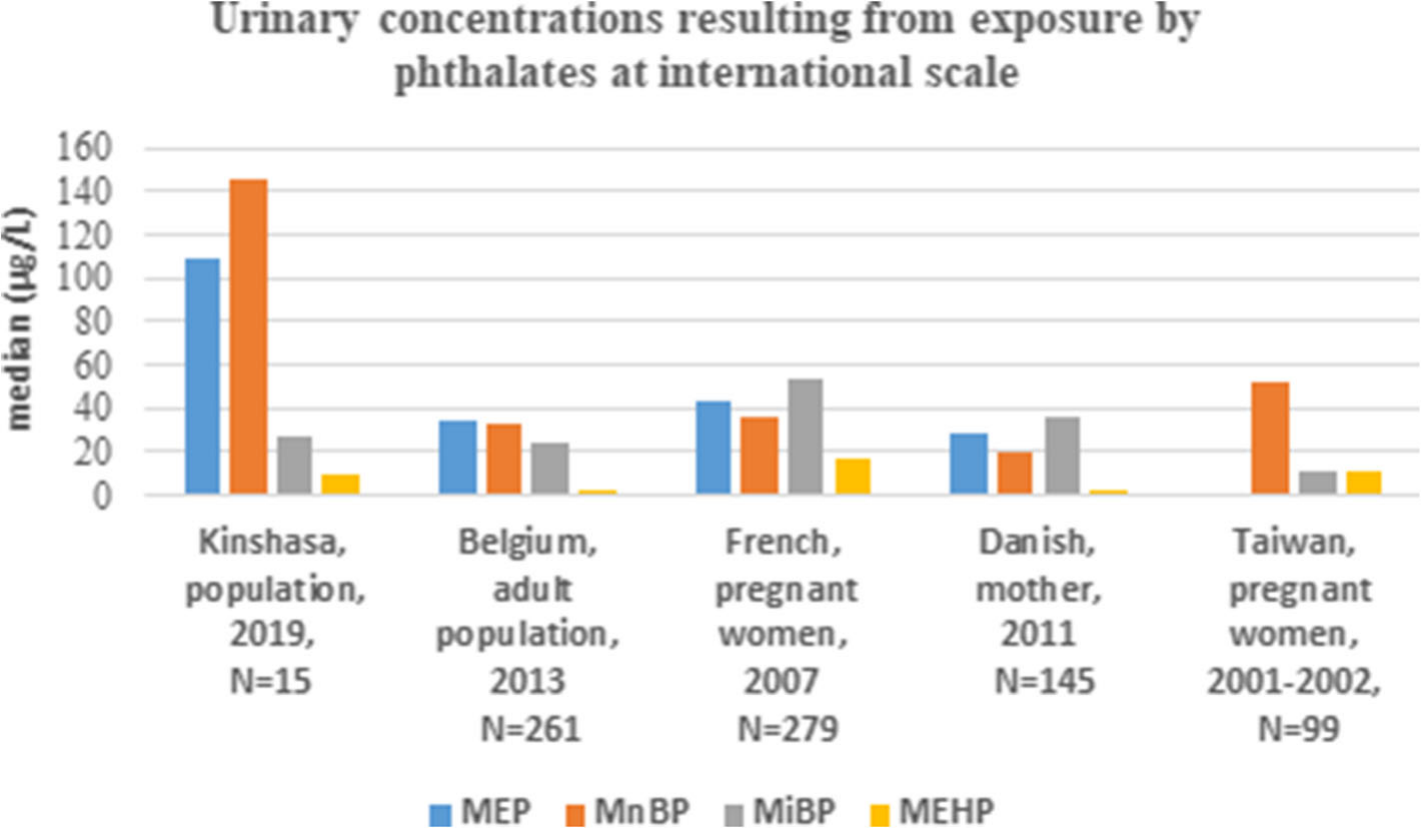
Urinary concentrations resulting from exposure by phthalates at international scale. **MEP**: Monoethyl Phthalate; **MnBP**: mono-n-butyl phthalate; **MiBP**: mono-iso-butyl phthalate; **MEHP**: mono-2-ethylhexyl phthalate

**Fig. 2:**
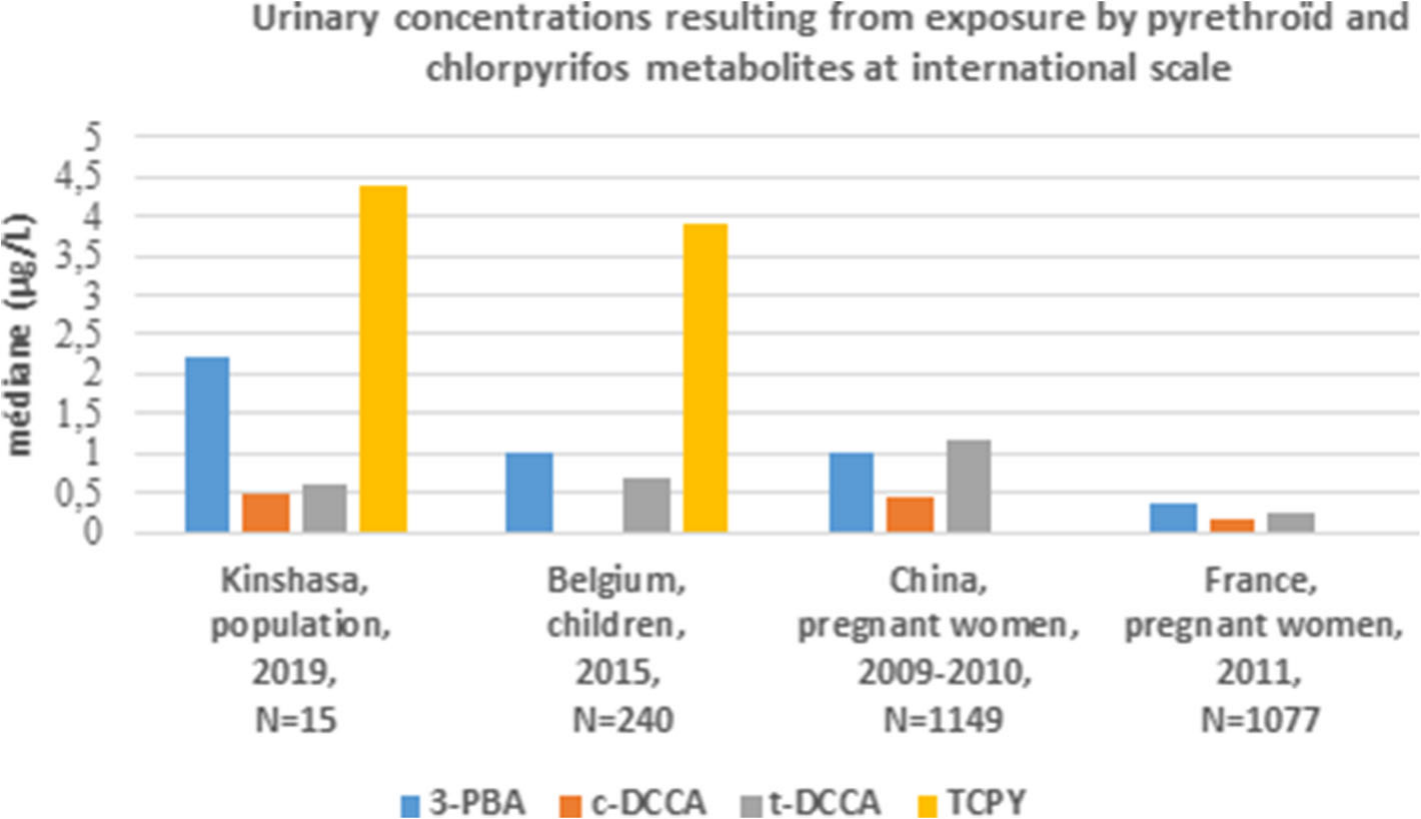
Urinary concentrations resulting from exposure by pyrethroïd and chlorpyrifos metabolites at international scale. **3-PBA**: 3-phenoxybenzoic acid**; c-DCCA**: cis-3-(2,2-dichlorovinyl)-2,2-dimethylcyclopropane-1-carboxylic acid; **t-DCCA**: trans-3-(2,2-dichlorovinyl)-2,2-dimethylcyclopropane-1-carboxylic acid; **TCPY**: 3,5,6-trichloro-2-pyridinol

All comparison results at both national and international scale are presented in Tables 3 and 4, and Figs. 1 and 2.

**Table 3:** Pollutants median values (μg/L) measured in urine, blood and serum from different worldwide populations

Pollutant	matrix	Year of collection	Population	Median	Reference
***Arsenic***	*Urine*				
		2012	Huelva (Spain), school children, *N* = 261	1.17	[22]
		2012–2013	Lubumbashi (DRC), pregnant women (control), *N* = 39	23.60^a^	[23]
		**2019**	**Kinshasa (DRC), population,** ***N*** **= 15**	**70.90**	**Current study**
***Cobalt***	*Urine*				
		2009	Ath-Belgium, men population, *N* = 52	0.16 ^a^	[24]
		2012–2013	Lubumbashi (DRC), pregnant women (control), *N* = 39	6.97 ^a^	[23]
		**2019**	**Kinshasa (DRC), population,** ***N*** **= 15**	**0.43**	**Current study**
***Cadmium***	*Urine*				
		2009	Ath-Belgium, men population, *N* = 52	0.21 ^a^	[24]
		2011–2012	Belgium, mother, *N* = 125	0.22	[7]
		2012	Huelva (Spain), school children, *N* = 261	0.29	[22]
		2012–2013	Lubumbashi (DRC), pregnant women (control), *N* = 39	0.60 ^a^	[23]
		**2019**	**Kinshasa (DRC), population,** ***N*** **= 15**	**0.61**	**Current study**
***Lead***	*Blood*				
		2009	Ath-Belgium, men population, *N* = 52	31.70 ^a^	[24]
		2011	Kinshasa (DRC), children 1–5 years, *N* = 100	86.00	[28]
		2012–2013	Lubumbashi (DRC), pregnant women (control), *N* = 39	50.80 ^a^	[23]
		2015–2016	Beijing, maternal blood, *N* = 156	17.60	[27]
		**2019**	**Kinshasa (DRC), population,** ***N*** **= 15**	**53.60**	**Current study**
***Glyphosate***	*Urine*				
		2012	German, adult population, *N* = 40	0.11	[26]
		2017	Irish, adult population, *N* = 50	0.87	[25]
		**2019**	**Kinshasa (DRC), population,** ***N*** **= 15**	**0.23**	**Current study**
***BP3***	*Urine*				
		2011	Danish, mother, *N* = 145	3.70	[30]
		2012	Tunisia, women, *N* = 34	1.73	[32]
		2013	Belgium, adult population, *N* = 261	1.30	[20]
		**2019**	**Kinshasa (DRC), population,** ***N*** **= 15**	**5.00**	**Current study**
***4,4’DDE***	*Serum*				
		2010–2011	Bolivian, women agriculture, *N* = 24	9.34	[42]
		2012–2013	South-Africa, pregnant women, *N* = 733	1.75^b^	[46]
		2013–2015	Lebanon, population, *N* = 314	0.13 ^b^	[45]
		2015	Belgium, adult population (women), *N* = 124	0.41	[1]
		**2019**	**Kinshasa (DRC), population,** ***N*** **= 15**	**1.46**	**Current study**
***PCB 153***	*Serum*				
		2005–2007	Anniston Community (USA), African-American, *N* = 353	1.42 ^b^	[47]
		2013–2015	Lebanon, population, *N* = 314	0.12 ^b^	[45]
		2015	Belgium, adult population, *N* = 251	0.36	[1]
		**2019**	**Kinshasa (DRC), population,** ***N*** **= 15**	**0.081**	**Current study**

^a^geometric mean

^b^ng /lipid weight concentrations converted to μg / L after multiplation by mean body lipid concentration (0.00735)

**Table 4:** Pollutants median values (μg/L) measured in urine and serum from different worldwide populations

Year of collection	Population	Parabens in urine			Perfluoroalkyl substances in serum		Alkylphosphates in urine		BPA in urine	TCS in urine	Reference
		MeP	EP	PrP	PFOS	PFOA	DEP	DETP			
2013	Belgium,adult,population, *N* = 261	16.1	1.7	1.2	–	–	–	–	–	–	[20]
2011	Danish, mother, *N* = 145	14	0.89	1.7	–	–	–	–	–	–	[30]
2010–2011	Spain, Young men, *N* = 215	17	1.8	0.7	–	–	–	–	–	–	[33]
2012	Tunisia, women, *N* = 34	34.94	1.77	3.06	–	–	–	–	–	–	[32]
2015–2018	Belgium, population, *N* = 237	–	–	–	3.61	1.6	–	–	–	–	[48]
2014–2016	USA,adolescents exposed, *N* = 118	–	–	–	3.72	1.8	–	–	–	–	[49]
2012	China, pregnant women, *N* = 141	–	–	–	4.31	3.95	–	–	–	–	[50]
2015	Czech,adult population, *N* = 300	–	–	–	2.43	0.76	–	–	–	–	[51]
2015	Belgium, children, *N* = 240	–	–	–	–	–	1.8	–	–	–	[15]
2006	Thailand, famers, *N* = 136	–	–	–	–	–	<LOQ	1.2	–	–	[37]
2012–2016	Jerusalem, Pregnant women, *N* = 273	–	–	–	–	–	2.72	0.55	–	–	[38]
2011	Belgium, population, *N* = 131	–	–	–	–	–	–	–	2.46	2.24	[17]
2009	Korea, adult population, *N* = 1870	–	–	–	–	–	–	–	2.07	1.53	[40]
2010–2011	Murcia (Spain),Young men, *N* = 215*	–	–	–	–	–	–	–	2.3	–	[41]
2012	Tunisia, women, *N* = 34	–	–	–	–	–	–	–	0.35	–	[32]
2011	Danish, mother, *N* = 145	–	–	–	–	–	–	–	2.1	0.64	[30]
**2019**	**Kinshasa (DRC), population,** ***N*** **= 15**	**445.39**	**0.47**	**31.35**	**0.5**	**0.482**	**0.87**	**<LOQ**	**1.36**	**40.13**	**Current study**

***BPA*** Bisphenol A, ***TCS*** Triclosan, ***MeP*** methylparaben, ***EP*** ethylparaben, ***PrP*** n-propylparaben, ***PFOS*** perfluoro-octane sulfonic, ***PFOA*** perfluoroctanoic acid, ***DEP*** diethylphosphate, ***DETP*** diethylthiophosphate

## Discussion

### Metals in urine

Except for Mn (detected at 20%), Sn and Sb (detected each at 47%), and Cd (detected at 93%), all other inorganic and metalloid compounds, investigated in urine were detected in all samples. Detected in most of the samples and with a median concentration of 70.91 μg/L, the population of Kinshasa is clearly more exposed to arsenic than populations in two other studies conducted in mining and industrial areas in Spain (Huelva) and in the DRC (Lubumbashi) (Table 3). Collected from 261 students, the first study reported a median content of 1.17 μg/L, while, the second study, based on 39 pregnant women (control population), stated a median concentration 3 times lower than here reported, probably due to an important food intake in Kinshasa, arising the necessity to figure out the exposure source and to identify the vulnerable population [22, 23]. With a median concentration of 0.61 μg/L, the cadmium exposure here reported seemed to be similar to those measured in the above-mentioned study (control population) from Lubumbashi [23], but two fold higher than the one reported in Spain, with a median content of 0.29 μg/L [22], but also than that stated in two Belgian studies, the first involving a population of 52 men of Ath with a geometric mean of 0.21 μg/L [24], and the second exploring 125 Belgian mothers reporting a median value of 0.22 μg/L [7] (Table 3).

### Glyphosate

Used in agriculture for crop protection, glyphosate, a widely used organophosphorus herbicide worldwide, is currently classified in Category 2A “probably carcinogenic to humans” by the International Agency for Research on Cancer (IARC) [25].

The glyphosate median outcome (0.23 μg/L) of the current study was far lower than that stated on 50 Irish adults with a median concentration of 0.87 μg/L [25]. But the study from Conrad A. et al. 2017 [26] on 40 German adults reported a lower median content (0.11 μg/L) than that stated here (Table 3). This result shows a clear glyphosate exposure in the investigated population of Kinshasa, due to a probable usage of this powerful herbicide by farmers or its possible presence in the various imported food products.

### Lead

Human exposure to lead (Pb) is caused by various industrial activities such as metallurgy, printing, ammunition, paintings, batteries of accumulators, etc. Atmospheric lead, which is largely responsible for lead body burden, is usually derived from gasoline products where it is used as an anti-detonator [14].

When compared to international studies conducted on 156 women in Beijing [27] and on 52 men in Belgium [24], with respective median content values of 17.6 μg/L and 31.7 μg/L, the median concentration here presented was higher (53.6 μg/L) (Table 3). These differences could probably be explained by a more important lead exposure source in Kinshasa, reinforcing the need to search for exposure source and to identify the vulnerable population.

In a national level, the observed median content was higher to the one of the control population from the 39 women study in Lubumbashi [23], reporting a median concentration of 50.8 μg/L, although the study of Tuakuila J. et al. 2013 [28] stated a higher median content of 86 μg/L on a population of 100 children recruited in 2011 in Kinshasa and aged from 1 to 5 years (Table 3). Based on a chronologic order of these publications, one can think of a possible lead exposure reduction, namely due to the sale suppression of leaded petrol throughout the country since 2009. But it is always important to maintain bio-surveillance studies as some parallel leaded petrol markets may still exist in Kinshasa. Moreover, the observed lead whole blood contents here reported could also be due to a possible release of lead into the blood from internal storage, indeed it is an element known to be accumulated in bones, soft tissues, and blood.

### Phthalates

For more than half a century, phthalates have been present in a wide range of daily products, so they are used as plasticizer, especially in polyvinyl chloride (PVC) and in cosmetics [20]. In the current study, median contents in MnBP and MEP, respectively 145.19 μg/L and 108.56 μg/L, were higher than those measured in 261 Belgian adults (33.3 μg/L of MnBP and 34.3 μg/L of MEP) [20], than those determined in 279 French pregnant women (35.7 μg/L of MnBP and 43.5 μg/L of MEP) [29], in 145 Danish women (20 μg/L of MnBP and 29 μg/L of MEP) [30], and in 99 Taiwanese women (52.39 μg/L of MnBP) [31]. This observation could probably be due to a strong presence of phthalates in plastic packaging used in Kinshasa, increasing the need for further investigations on the exposure source and the identification of susceptible population. A slightly lower median concentration in MiBP was observed when compared to the French and Danish studies as well as the median content in MEHP was slightly lower than those reported in the French and Taiwanese studies (Fig.1).

### Parabens

With bactericidal and fungicidal properties, parabens are largely used in cosmetics, food, and pharmaceuticals as preservatives. Detected in all investigated samples, median concentrations of MeP (445.39 μg/L) and PrP (31.35 μg/L) were clearly higher than those reported in 34 Tunisian women (34.94 μg/L of MeP and 3.06 μg/L of PrP) [32], 215 young Spanish (17 μg/L of MeP and 0.7 μg/L of PrP) [33], 145 Danish women (14 μg/L of MeP and 1.7 μg/L of PrP) [30], and 261 Belgian adults (16.1 μg/L of MeP and 1.2 μg/L of PrP) [20], probably due to a greater use of these preservatives in cosmetics, food and pharmaceutical products marketed in Kinshasa. Thereby, further investigations are needed to detect products containing parabens, their corresponding quantities and identify the vulnerable population. In contrast, this study has found a lower median concentration in EP (0.47 μg/L) (Table 4).

### Benzophenone-3 (BP3)

Added in cosmetics and food packaging, benzophenones have ultraviolet filters properties, reducing their deleterious effects on the skin or food. Detected in all investigated samples, the current study has found a median concentration in BP3 (5 μg/L) higher than those reported in the Belgian adult population (1.3 μg/L) [20], in Tunisian women (1.73 μg/L) [32], and in Danish women (3.7 μg/L) [30], probably due to the presence of this UV filter in cosmetics or on food packaging, reinforcing the need to identify the source, used concentrations, and the vulnerable population (Table 3).

### Pyrethroids and dialkylphosphate pesticides

Pyrethroids and dialkyl-phosphates (DAPs) are among pesticides largely used in crop culture for the protection of harvests and in public health for the fight against diseases vectors. Associated with weak enzyme baggage, the immaturity in the development of some organs makes children around the world among vulnerable populations to environmental pollution [34].

In the current study, median contents in TCPY (4.4 μg/L), in 3-PBA (2.23 μg/L), and in c-DCCA (0.47 μg/L) were higher than those measured in 240 Belgian children (with 1 μg/L of 3-PBA and 3.9 μg/L of TCPY) [15], in 1149 Chinese pregnant women (with 1.01 μg/L of 3-PBA and 0.44 μg/L of c-DCCA) [35], and in 1077 French pregnant women (with 0.36 μg/L of 3-PBA and 0.16 μg/L of c-DCCA) [36]. Only the median concentration in t-DCCA was lower than those on the two first studies. The high concentration observed in the present study was probably due to a very high use of pyrethroid insecticides in mosquito nets, insecticide sprays and other products for agricultural use (Fig.2). With a detection frequency of 53% for DEP and 47% for DETP with respective median contents values of 0.87 μg/L and < 0.5 μg/L, this study has presented a slightly lower DAPs exposure when compared to studies on 240 Belgian children (with 1.8 μg/L of DEP) [15], on 136 Thai farmers (with DEP median value <LOQ and 1.2 μg/L of DETP) [37], and on 273 pregnant women in Jerusalem (with 2.72 μg/L of DEP and 0.55 μg/L of DETP) [38] (Table 4).

### Bisphenols and triclosan

Used as epoxy resin monomers, bisphenols are aromatic organic compounds used in the manufacture of plastics and poly epoxides. Added in toothpastes and cosmetics for its antibacterial properties, triclosan is likewise suspected to be endocrine disruptor for instance, it is suspected to be associated with the decrease of some biomarkers of thyroid function [39].

The median content in triclosan here observed (40.13 μg/L) was one or two orders of magnitude higher than those observed in a Belgian population with 131 participants (2.24 μg/L) [17], in 1870 Korian adults (1.53 μg/L) [40], and on 145 Danish women (0.64 μg/L) [30]. Conversely, the investigated population of Kinshasa presented a median concentration in bisphenol A (1.36 μg/L) lower than those reported in the above studies and that on 215 young Spanish of Murcia (2.3 μg/L) [41], but higher than that on 34 Tunisian women (0.35 μg/L) [32] (Table 4). The current study showed that the population of Kinshasa was also exposed to the new bisphenols, especially to bisphenol F (detected in 67% of the samples) and bisphenol S (detected in 80% of the samples) (Table 1). The exposure in the population of Kinshasa to these pollutants is probably due to the presence of triclosan in cosmetic products, toothpastes and that of bisphenols in cans and plastics sold in the city.

### Chlorinated pesticides

Previously used in the fight against pests in agriculture, the p,p’-dichlorodiphenyltrichloroethane (DDT) is still used in many tropical and subtropical regions to fight against mosquitoes vector of malaria [42]. In DRC, the use of organochlorine compounds has been banned since March 2005 but Nuapia Y. et al. 2016 [43] found these compounds in raw foods (beans, cabbage, fish and beef) sold in Kinshasa.

Main metabolite of DDT in the environment and in the living organism, p,p’- dichlorodiphenyldichlorethylene (p,p’-DDE) is considered as a marker of previous exposure [44]. Detected in 87% of the samples with a median content value of 1.46 μg/L, the investigated population in Kinshasa presented a concentration largely higher than those on 124 Belgian women (median = 0.41 μg/L) [1] and on Lebanese population with 314 participants (median = 0.13 μg/L) [45], probably either due to a remobilization of DDT accumulated in the soil, a late ban of DDT in DRC compared to other countries or the existence of an illegal DDT market in Kinshasa. Studies on 733 South African pregnant women and on 24 Bolivian women farmers reported a higher median concentration [42, 46] (Table 3).

### Polychlorinated biphenyls

Used as insulating fluid in electrical equipment and additives in putty, polychlorinated biphenyls (PCB) are POPs as they also remain intact in the environment for many years. Comparing median contents in PCB 153 of studies performed on 251 Belgians (0.36 μg/L) [1], 314 Lebanese (0.12 μg/L) [45], and on 353 Afro-Americans (1.42 μg/L) [47], the investigated population in Kinshasa was weakly exposed to these compounds (median concentration in the present study = 0.081 μg/L), probably due to their low use in the Kinshasan market (Table 3).

### Perfluoroalkyl substances

Surfactants with high thermal stability, perfluoroalkyl substances (PFAS) are found in various everyday products (kitchen utensils, stoves, microwave packaging, raincoats, sportswear, etc.) thanks to their waterproofing and non-stick properties [8].

In the current study, a low contamination in PFAS (for instance, median PFOS = 0.5 ng/mL and median PFOA = 0.48 ng/mL) was observed in the population comparatively to studies on 237 Belgians (with median concentration of 3.61 μg/L for PFOS and 1.6 μg/L for PFOA) [48], 118 American teenagers (median PFOS = 3.72 ng/mL and median PFOA = 1.8 ng/mL) [49], 141 Chinese pregnant women (median PFOS = 4.31 ng/mL and median PFOA = 3.95 ng/mL) [50], and on 300 Czech adults (median PFOS = 2.43 ng/mL and median PFOA = 0.76 ng/mL) [51], reflecting a probable low presence of these compounds in the market of Kinshasa (Table 4).

### Phenolic organohalogens

Contamination by phenolic organohalogens (POH) seemed to be weak in the investigated population: pentachlorophenol, a pesticide used namely in the protection of timber, was only detected in 14% of the population, while it was present in 100% of 272 Belgian volunteers as reported by Dufour et al. 2017 [52], with a median content value of 593 pg/mL (both populations were explored with the same analytical method). Likewise, weak was the contamination by OH-CBs, metabolites of PCBs. Only 4-OH-CB 187 was found in more than 50% of individuals (57%), with a median concentration of 2.4 pg/mL while Dufour et al. 2017 [52] highlighted a detection frequency of 100% and a median level of 39.4 pg/mL in the Belgian population, reflecting a reduced contamination observed for PCBs in the investigated population of Kinshasa.

### Brominated flame retardants

Finally, brominated flame retardants (BFR) are compounds incorporated in many materials (especially plastics) to increase their fire resistance and to help reducing the risk of fire. They are therefore found in many everyday products, including vehicles, clothes, furniture, electronic equipment, etc. The investigated population of Kinshasa seemed to escape to these compounds as the most frequent substances, PBDE 47 and 153 were only detected in 27% of the samples, with maximum concentrations of 15 and 13 pg/mL, respectively. This situation is close to that observed in Belgium, with detection frequencies lower than 40% for all PBDEs highlighted with the same analytical method [48], but largely far from the contamination observed in the USA, where diverse studies showed median levels in total PBDEs ranging from 10 to values higher than 40 ng/g in lipids or approximatively 73.5 pg/mL and 294 pg/mL [53].

### Future large scale studies

This pilot study brings us several lessons. First, it is difficult to collect blood samples in the population of Kinshasa. Indeed, because of spiritual considerations or superstition, many people we asked to participate to the study refuse to give blood sample. This element should be considered when writing the final protocol for larger scale study: blood sample collection will be associated with important difficulties, waste of time and could biased the participant recruitment by potentially excluding some socio-economic profiles from the population. Secondly, the profile of pollutant contamination observed in the Kinshasa’s population is highly different from those highlighted in Western countries as like Belgium.

Considering these two parameters, pollutants families could be classified by order of priority. In the group of “high priority” pollutants, we included As, triclosan and parabens, these pollutants were measured in urine at levels more than 10-fold higher than those observed in other general populations. Considering the high exposition levels observed for these compounds, they should be included in future investigations. Phthalates and BP3 are quantified with the same analytical method than parabens and BPs are measured simultaneously to triclosan. Consequently, these pollutants could be added to the high priority group without additional costs. The second group, “intermediate priority” pollutants groups gathers glyphosate, pyrethroids and dialkylphosphate pesticides that were measured at levels similar to those observed in other populations. Lead and OCPs were also added to the “intermediate priority” group, the contamination determined in our population for these pollutants was relatively high but they are measured in blood and are thus more difficult to investigate. The pollutants of this second group will be included or not in further studies according to the available resources. In the last group: “low priority” pollutants, we included PFASs, POHs, PCBs and BFRs, these compounds were measured in low concentrations in our population compared to Western countries and require blood samples, thus they probably will not be included in large scale study in Kinshasa.

The recruitment is also an important parameter to consider during the redaction of the protocol. In order to avoid spiritual considerations, religion communities and opinion leaders should be involved in the recruitment process, this should allow an optimal sensitization and recruitment of study population. Concerning the study cohort composition, we consider two options. First, we could design our study protocol to explore the whole general population. The recruitment will be targeted in order to recruit a similar number of individuals for each age group (18–29 years, 30–39 years, 40–49 years, 50–59 years, > 60 years) and gender group, children could also be included in the protocol (age classes: < 6 years, 6–12 years, 12–18 years). The second option is to focus the recruitment on vulnerable individuals i.e. children, adolescents, and women of childbearing age. Indeed, the childhood and the puberty are associated with important developmental changes and environmental pollutants could interfere with this physiological process, especially endocrine disruptors. In the same way, contamination during in utero life could be associated with important long-term consequences, therefore the exploration of women of childbearing age is of primary importance [2].

## Conclusion

In conclusion, thanks to the information collected during this preliminary study, 3 protocols could be considered:

- The “minimal” protocol: for each age group (18–29 years, 30–39 years, 40–49 years, 50–59 years and > 60 years), 5 women and 5 men will be randomly (notwithstanding the profession) selected. To recruit volunteers, announcement will be made on the local radio and during the religious services at the church. Each volunteer will be asked to provide urine sample and to complete questionnaire concerning his demographics and life habits. In urine samples, we will measure the concentrations of As, triclosan, bisphenols, phthalates and parabens.
- The “intermediate” protocol: recruitment will be the same as in “minimal” protocol but in addition, we will determinate in urine samples the concentrations of glyphosate, pyrethroids and dialkylphosphate pesticides. We will also ask to the volunteers to provide blood samples in order to estimate the contamination by lead and OCP. Blood samples collection is difficult, some effort will be necessary and contacts will have to be established with community and religious leaders to try to overcome spiritual and superstitious fears.
- The “optimal” protocol: modalities of the “intermediate” protocol (by excluding the collection of blood samples) will be extended to children. Ten girls and 10 boys will be randomly selected by age group (< 6 years, 6–12 years, 12–18 years). In additions, 30 women in childbearing age (18–29 years) will be recruited (for urinary and blood exploration).

The protocol chosen for the larger scale study will depend on the resources collected to perform these investigations, number of individuals recruited in each age group could be adjusted if resource are sufficient. The present report clearly indicates that the population of Kinshasa is not spared by the investigated environmental pollutants. Therefore, it is of paramount importance to scale-up and validate this study to a larger population of Kinshasa to obtain a database on pollutants and identify potential hot spots of exposure, in order to establish relationship between certain socio-demographic characteristics (age, sex, food, smoking, professional activity, etc.) and the level of exposure to pollutants, to investigate possible sources of exposure and to explore potential associations between the contamination and the prevalence of some chronic diseases in the population of Kinshasa.

## Supplementary Information

**Additional file 1.** : Supplementary materials .

## Data Availability

The datasets used and/or analysed during the current study are available from the corresponding author on a reasonable request.

## Abbreviations

DRC: Democratic Republic of Congo
POPs: Persistent organic pollutants
OCP: Organochlorine pesticides
PCB: Polychlorinated biphenyls
BFR: Brominated flame retardants
POH: Phenolic organo-halogens
PFAS: Perfluoroalkyl substances
FMOC: Fluorenylmethoxycarbonyl chloride
c- and t-DCCA: cis- and trans-3-(2,2-dichlorovinyl)-2.2-dimethylcyclopropane carboxylic acid
3-PBA: 3-phenoxybenzoic acid
4F-3-PBA: 4-fluoro-3-phenoxybenzoic acid
DBCA: 3-(2,2-dibromovinyl)-2.2-dimethylcyclopropane carboxylic acid
TCPY: 3,5,6-trichloro-2-pyridinol
MTBSTFA: N-tert-butyldimethylsilyl-N-methyltrifluoroacetamide
DAPs: Dialkylphosphates
DMTP: Dimethylthiophosphate
DMDTP: Dimethyldithiophosphate
DEP: Diethylphosphate
DETP: Diethylthiophosphate
DEDTP: Diethyldithiophosphate
SPE: Solid phase extraction
MEP: Monoethyl phthalate
MiBP: Mono-iso-butyl phthalate
MnBP: Mono-n-butyl phthalate
MBzP: Monobenzyl phthalate
MEHP: Mono-2-ethylhexyl phthalate
5-OH-MEHP: Mono-2-ethyl-5-hydroxyhexyl phthalate
5-oxo-MEHP: Mono-2-ethyl-5-oxohexyl phthalate
MeP: Methylparaben
EP: Ethylparaben
PrP: n-propylparaben
BP: n-butylparaben
BP-3: Benzophenone-3
TCS: Triclosan
BP: Bisphenols
HCB: Hexachlorobenzene 4;4′-DDE 4,4′-dichlorodiphenyl-dichloroethylene
PBDEs: Polybrominated diphenylethers
PFOS: Perfluoro-octane sulfonic
PFOA: Perfluoroctanoic acid
PFHxS: Perfluorohexane sulfonate
PFNA: Perfluorononanoic acid
PFDA: Perfluorodecanoic acid
PFHpA: Perfluoroheptanoic acid
PFUdA: Perfluoroundecanoic acid
PCP: Pentachlorophenol
TBBPA: Tetrabromobisphenol A
2,4,6-TBP: 2,4,6-tribromophenol
2,3,6-TBP: 2,3,6-tribromophenol
2,4,5-TBP: 2,4,5-tribromophenol 2,3,4,6-TeBP 2,3,4,6-tetrabromophenol
6-OH-BDE 47: 6-hydroxy-polybromodiphenylether 47
5-OH-BDE 47: 5-hydroxy-polybromodiphenylether 47
5′–OH–BDE 99: 5′-hydroxy-polybromodiphenylether 99
4-OH-CB 107: 4-hydroxy-polychlorinated biphenyl 107
3-OH-CB 138: 3-hydroxy-polychlorinated biphenyl 138
4-OH-CB 146: 4-hydroxy-polychlorinated biphenyl 146
3-OH-CB 153: 3-hydroxy-polychlorinated biphenyl 153
4-OH-CB 172: 4-hydroxy-polychlorinated biphenyl 172
3-OH-CB 180: 3-hydroxy-polychlorinated biphenyl 180
4-OH-CB 187: 4-hydroxy-polychlorinated biphenyl 187
LOQ: Limit of Quantification
DF: Detection Frequency
IARC: International Agency for Research on Cancer
