# Supplementary material for: Bio-surveillance of environmental pollutants in the population of Kinshasa, Democratic Republic of Congo (DRC): a small pilot study
Source: Arch Public Health. 2021 Nov 15;79:197. doi: 10.1186/s13690-021-00717-x (PMC8591802; doi:10.1186/s13690-021-00717-x)
Supplement: Supplementary file 1 — Additional file 1. : Supplementary materials . [file 13690_2021_717_MOESM1_ESM.docx]

**SUPPLEMENTARY MATERIALS**

**Analysis of metals and metalloids in urine**

Inorganic compounds in urine were quantified using an inductively coupled plasma mass spectrometer (ICP-MS) 7700 series coupled to an autosampler ASX-500 series (both from Agilent Technologies). The nebulizer was Babington type. Two different sample preparations were used. The first was used for the determination of the compounds commonly present in human urine at low levels (Bi, Cd, Cr, Mn, Ni, Sb, Sn, Tl and V): urine sample (500 µL) was mixed with 2500 µL of an aqueous solution of nitric acid (0.5%) and 100 µL of a solution of internal standard (Rh, Sc and Ge at 50 µg/L in nitric acid aqueous solution (0.5%)). The second method was used to measure the levels of the compounds commonly present in human urine at higher levels (As, Cu, Mo, Se and Zn): urine sample (400 µL) was mixed with 4600 µL of an aqueous solution of nitric acid (0.5%) and 100 µL of a solution of internal standard (Rh, Sc and Ge at 500 µg/L in nitric acid aqueous solution (0.5%)). Diluted samples were injected on the ICP-MS for analysis through a peristaltic pump. Ion monitored by MS, specific internal standard and range of concentrations measured for each compounds were gathered in table S1. The radio frequency power of the ICP torch was set at 1550 W, argon was used as nebulizer gas and plasma gas at the flow of 1.2 L/min and 15 L/min, respectively.

| Compounds | Ion monitored (m/z) | Internal standard | Range of concentrations (µg/L) |
| --- | --- | --- | --- |
| *Low levels* |  |  |  |
| Bi | 209 | Rh | 0.12-98 |
| Cd | 111 | Rh | 0.12-98 |
| Cr | 52 | Sc | 0.23-98 |
| Mn | 55 | Sc | 0.89-98 |
| Ni | 60 | Ge | 1.63-98 |
| Sb | 121 | Rh | 0.14-98 |
| Sn | 118 | Rh | 0.73-98 |
| Tl | 205 | Rh | 0.09-98 |
| V | 51 | Sc | 0.18-98 |
| *High levels* |  |  |  |
| As | 75 | Ge | 0.14-2000 |
| Cu | 63-65 | Ge | 1.6-2000 |
| Mo | 95 | Rh | 5.0-2000 |
| Se | 78 | Ge | 7.9-2000 |
| Zn | 66 | Ge | 15-2000 |
| *Internal standards* |  |  |  |
| Rh | 103 |  |  |
| Sc | 45 |  |  |
| Ge | 72 |  |  |

Table S1: Ion monitored by MS, specific internal standard and range of concentrations measured for each compound.

**Analysis of glyphosate in urine**

Fifty µL of internal standard solution (glyphosate ^13^C_2_ ^15^N at 0.05 mg/L), 1 mL of borate buffer 5% and 3 mL of solution the derivatizing reagent 9-fluorenylmethylchloroformate chloride (FMOC) at 20 g/L in acetone were mixed with 1 mL of urinary sample. The mixture was allowed to react overnight at room temperature and protected from light. The derivatizated sample was then evaporated under a gentle stream of nitrogen (at max. 40°C) in order the remove the acetone from the mixture. Five mL of ethyl acetate were then added to the aqueous residue and vortexed for 10 min. After centrifugation (5 min at 3000 rpm), the organic phase containing residual FMOC and other apolar compounds was discarded. Then, the aqueous phase was acidified by the addition of 100 µL of concentrated hydrochloric acid (37%) and 5 mL of diethylether were added, the mixture were vortexed during 10 min, then centrifugated (5 min at 3000 rpm) and the organic phase was collected. This process was repeated once and both organic layers were combined and evaporated to dryness under a gentle stream of nitrogen at 40°C. Residue was reconstituted in 100 µL of a solution of 5 mM ammonium acetate in water (pH adjusted to 4.8 with acetic acid 1 M) and acetonitrile (80/20, v/v) and transferred into a vial before LC-MS/MS analysis.

The chromatographical separation was performed using an Acquity Ultra Performance UHPLC system (Waters, Milford, MA, USA) equipped with an Acquity UHPLC BEH C18 column (2.1 × 50 mm, 1.7 µm) from Waters (Milford, MA, USA). The column temperature was set at 40°C. The mobile phases consisted in 5 mM ammonium acetate in water (pH adjusted to 4.8 with acetic acid 1 M) (phase A) and acetonitrile (phase B). The injection volume was 10 µL, the mobile phase flow was set at 0.4 mL/min and the gradient conditions were summarized in Table S2.

| Time (min) | % of mobile phase A | % of mobile phase B |
| --- | --- | --- |
| 0.00 | 90 | 10 |
| 1.00 | 90 | 10 |
| 5.00 | 75 | 25 |
| 5.50 | 0 | 100 |
| 6.50 | 0 | 100 |
| 6.65 | 90 | 10 |
| 8.00 | 90 | 10 |

Table S2: mobile phases’ gradient.

The identification and the quantification of the glyphosate was performed using a Xevo TQS (Waters, Milford, MA, USA). The mass spectrometer operated in positive electrospray ionization (capillary voltage = 3 kV). The source and the desolvatation temperatures were set at 150°C and 350°C, respectively. Nitrogen was used as cone and desolvatation gas (flow set at 50 L/h and 800 L/h, respectively) and argon was used as collision gas at 0.15 mL/min. MRM transitions, cone voltage (CV), collision energy (CE) and dwell times for glyphosate and internal standard were reported in table S3. The limit of quantification was 0.08 ng/mL.

| Compounds | MRM (m/z) | CV (V) | CE (eV) | Dwell time (s) |
| --- | --- | --- | --- | --- |
| Glyphosate | **392 🡪 87.6** | **21** | **20** | **0.027** |
|  | 392 🡪 213.8 | 21 | 10 | 0.027 |
| Glyphosate ^13^C_2_ ^15^N | 395 🡪 90.6 | 21 | 20 | 0.027 |
|  | **395 🡪 216.8** | **21** | **10** | **0.027** |

Table S3: MRM transitions, cone voltage (CV), collision energy (CE) and dwell times for glyphosate and internal standard. MRM transitions used for the quantification are in bold.

**Analysis of triclosan and bisphenols in urine**

Twenty microliters of internal standard solution (BPA-d_14_, BPS-d_8_ and triclosan-^13^C_12_ at 1 mg/L) were added to 3 mL of urine sample. Mixture was submitted to hydrolysis with β-glucuronidase and sulfatase in sodium acetate buffer (1M, pH = 4.5) for 30 min at 40°C. The hydrolysis reaction was stopped by the addition of 200 µL of formic acid and the sample was then extraction on solid phase extraction (SPE) cartridge after sonication (15 min) and centrifugation (3000 rpm during 15 min). SPE was performed with an Oasis HLB cartridge (3cm³, 60 mg, Waters). The cartridge was previously conditioned and washed with 3 mL of dichloromethane, 2 × 3 mL of methanol and 3 mL of water. After the sample loading, the cartridge was washed with 3 mL of water and then centrifugated at 5000 rpm during 5 min to completely dry the solid phase. Analytes were then eluted with 2 × 2.5 mL of a mixture methanol/dichloromethane (1/1, v/v). The eluate was then evaporated to dryness under a gentle stream of nitrogen at 30°C. Residue was reconstituted in 1 mL of water and 50 µL of KOH 2 M and extracted with 3 mL of ethyl acetate. The organic phase was collected and evaporated to dryness under a gentle stream of nitrogen at 30°C. Residue was reconstituted in 30 µL of ethyl acetate and 20 µL of derivatizating reagent N-Methyl-N-(trimethylsilyl)trifluoroacetamide (MSTFA) and transferred into a vial. Extract was analyzed by gas chromatography coupled to mass spectrometer (GC-MS/MS).

An Agilent 7890A GC/7000A GC Triple Quad mass spectrometer (Agilent Technologies,California, USA) equipped with an Agilent HP-5MS capillary column (30 m × 0.25 mm i.d. × 0.25 µm internal film thickness) was used for the GC-MS/MS analysis. The injector operated in pulsed splitless mode (50 psi for 1.25 min) at 250 °C. Helium was used as carrier gas, the flow was constant and set at 1.23 mL/min. The temperature gradient was set as follows: the initial temperature was 70°C held for 1.25 min, then increase to 210°C at the rate of 75°C/min, then to 250°C at the rate of 7°C/min and finally to 325°C at the rate of 20°C/min, the final temperature was held for 3 min.

Compounds were analyzed with mass spectrometer operating in Multiple Reaction Monitoring (MRM), with electronic impact (EI) source (electronic energy: -70 eV). The temperatures of the transfer line, the source and the quadrupoles were set at 250°C, 230°C and 150°C respectively. The transitions monitored, the internal standard and the collision energy used for each analyte were gathered in table S4.

| Compounds | MRM transition (m/z) | Collision energy | Internal standard | LOQ (ng/mL) |
| --- | --- | --- | --- | --- |
| BPAF | 411 🡪 73 | 33 | BPA-d_14_ | 0.063 |
|  | **480 🡪 411.1** | **14** |  |  |
| BPF | 343.9 🡪 73 | 36 | BPA-d_14_ | 0.074 |
|  | **343.9 🡪 179** | **22** |  |  |
| BPA-d_14_ | **368 🡪 73** | **39** |  |  |
|  | 368 🡪 197 | 26 |  |  |
| BPA | 356.9 🡪 73 | 39 | BPA-d_14_ | 0.293 |
|  | **356.9 🡪 191.1** | **21** |  |  |
| BPZ | 368.9 🡪 73 | 32 | BPA-d_14_ | 0.057 |
|  | **368.9 🡪 203** | **14** |  |  |
| BPS-d_8_ | **401.7 🡪 73** | **39** |  |  |
|  | 386.7 🡪 73 | 36 |  |  |
| BPS | 394 🡪 73 | 39 | BPS-d_8_ | 0.093 |
|  | **379 🡪 73** | **34** |  |  |
| BPAP | 418.9 🡪 73 | 37 | BPA-d_14_ | 0.211 |
|  | **433.9 🡪 419.2** | **14** |  |  |
| BPP | 474.9 🡪 73 | 39 | BPA-d_14_ | 0.091 |
|  | **474.9 🡪 207.1** | **26** |  |  |
| Triclosan-^13^C_12_ | **371.7 🡪 206.1** | **20** |  |  |
|  | 358.8 🡪 206.1 | 20 |  |  |
| Triclosan | **359 🡪 200** | **15** | Triclosan-^13^C_12_ | 0.2 |
|  | 344.7 🡪 200 | 15 |  |  |

Table S4: MRM transitions monitored, internal standard and energy collision used for the analysis of bisphenols and triclosan. MRM transitions used for the quantification are in bold.

**Analysis of lead in whole blood**

The concentration of lead in whole blood was determined using an inductively coupled plasma mass spectrometer (ICP-MS) 7700 series coupled to an autosampler ASX-500 series (both from Agilent Technologies) and the nebulizer was Babington type. Blood sample (500 µL) was mixed with 4500 µL of a solution of nitric acid (0.5%), n-butanol (0.2%) and triton (0.1%) in water and 100 µL of solution of internal standard (Rh, Sc and Ge at 500 µg/L in nitric acid aqueous solution (0.5%)). Diluted sample was injected on the ICP-MS for analysis through a peristaltic pump. The ion monitored for lead was 208 (m/z), the specific internal standard was Rh, the lower and the upper limit of quantification were 0.5 µg/L and 480 µg/L, respectively. The radio frequency power of the ICP torch was set at 1550 W, argon was used as nebulizer gas and plasma gas at the flow of 1.2 L/min and 15 L/min, respectively.

Mean, geometric mean, median and range concentrations for all investigated molecules are detailed in the following table S5.

|  | Pollutant | Mean | Geometric mean | Median | Minimum | Maximum |
| --- | --- | --- | --- | --- | --- | --- |
| ***Urinary*** |  |  |  |  |  |  |
|  | Be | ‹LOQ (‹LOQ) | ‹LOQ (‹LOQ) | ‹LOQ (‹LOQ) | ‹LOQ (‹LOQ) | ‹LOQ (‹LOQ) |
|  | V | 0.38 (0.14) | 0.32 (0.13) | 0.36 (0.13) | ‹LOQ (‹LOQ) | 0.99 (0.25) |
|  | Cr | 0.66 (0.26) | 0.55 (0.23) | 0.45 (0.21) | 0.24 (0.12) | 1.49 (0.66) |
|  | Mn | ‹LOQ (‹LOQ) | ‹LOQ (‹LOQ) | ‹LOQ (‹LOQ) | ‹LOQ (‹LOQ) | 2.57 (0.76) |
|  | Ni | 9.81 (3.69) | 7.99 (3.41) | 7.28 (3.83) | 2.94 (1.33) | 35.92 (6.76) |
|  | Sn | 0.78 (0.37) | 0.76 (0.33) | ‹LOQ (‹LOQ) | ‹LOQ (‹LOQ) | 1.23 (1.03) |
|  | Sb | 0.16 (0.06) | ‹LOQ (‹LOQ) | ‹LOQ (‹LOQ) | ‹LOQ (‹LOQ) | 0.55 (0.09) |
|  | Pt | ‹LOQ (‹LOQ) | ‹LOQ (‹LOQ) | ‹LOQ (‹LOQ) | ‹LOQ (‹LOQ) | ‹LOQ (‹LOQ) |
|  | Tl | 0.58 (0.23) | 0.50 (0.21) | 0.44 (0.22) | 0.16 (0.11) | 1.13 (0.44) |
|  | Bi | 0.25 (0.13) | 0.25 (0.11) | 0.24 (0.09) | 0.22 (0.03) | 0.31 (0.41) |
|  | Cu | 28.06 (10.73) | 22.37 (9.55) | 19.28 (8.90) | 8.76 (5.66) | 51.40 (28.80) |
|  | Zn | 1309 (399) | 755 (322) | 667 (275) | 85.45 (151) | 7099 (1201) |
|  | Se | 58.99 (19.56) | 44.85 (19.16) | 43.09 (18.21) | 8.78 (14.36) | 191.65 (28.56) |
|  | Mo | 94.89 (33.77) | 66.85 (28.56) | 76.07 (30.85) | 14.42 (4.58) | 294.22 (71.50) |
|  | Cobalt | 1.0 (0.31) | 0.57 (0.24) | 0.43 (0.21) | 0.16 (0.089) | 6.11 (0.87) |
|  | Cadmium | 1.15 (0.33) | 0.66 (0.28) | 0.61 (0.26) | ‹LOQ (‹LOQ) | 6.12 (0.87) |
|  | Arsenic | 81.74 (32.33) | 69.73 (29.79) | 70.91 (30.86) | 32.12 (11.97) | 215.32 (57.28) |
|  | Glyphosate | 0.22 (0.095) | 0.19 (0.083) | 0.23 (0.098) | 0.09 (0.05) | 0.40 (0.18) |
|  | MeP | 699.98 (335.48) | 216.12 (92.32) | 445.39 (121.96) | 15.06 (9.94) | 4467.5 (1386.5) |
|  | EP | 11.94 (2.96) | 0.73 (0.31) | 0.47 (0.25) | ‹LOQ (‹LOQ) | 86.5 (26.83) |
|  | PrP | 290.62 (157.53) | 38.96 (16.64) | 31.35 (5.35) | 0.97 (0.65) | 2509.15 (778.75) |
|  | MEP | 309.94 (96.09) | 113.09 (48.31) | 108.56 (41.96) | 13.96 (9.45) | 2366.9 (734.6) |
|  | MEHP | 16.47 (6.26) | 8.53 (3.64) | 9.03 (3.25) | 0.97 (0.84) | 62.45 (15.75) |
|  | MnBP | 229.67 (92.86) | 176.42 (75.36) | 145.19 (60.72) | 32.05 (31.79) | 638.11 (292.3) |
|  | MiBP | 31.39 (13.42) | 25.26 (10.79) | 26.52 (9.32) | 7.43 (4.1) | 72.37 (27.97) |
|  | MBzP | 2.35 (0.85) | 1.72 (0.73) | 1.90 (0.87) | ‹LOQ (‹LOQ) | 9.59 (1.99) |
|  | BP3 | 6.76 (2.59) | 4.97 (2.12) | 5.00 (1.93) | 0.76 (0.65) | 23.31 (6.67) |
|  | c-DCCA | 0.81 (0.28) | 0.41 (0.18) | 0.47 (0.23) | ‹LOQ (‹LOQ) | 3.9 (0.69) |
|  | t-DCCA | 1.34 (0.47) | 0.72 (0.31) | 0.59 (0.31) | ‹LOQ (‹LOQ) | 0.17 (0.12) |
|  | TCPY | 37.68 (13.52) | 9.19 (3.92) | 4.43 (2.19) | 0.40 (0.44) | 123.36 (54.6) |
|  | 3-PBA | 16.88 (4.84) | 3.36 (1.44) | 2.25 (1.22) | 0.29 (0.15) | 173.20 (48.50) |
|  | DBCA | 2.21 (0.95) | 1.62 (0.69) | 1.93 (0.71) | ‹LOQ (‹LOQ) | 5.93 (2.99) |
|  | FPBA | ‹LOQ (‹LOQ) | ‹LOQ (‹LOQ) | ‹LOQ (‹LOQ) | ‹LOQ (‹LOQ) | 0.18 (0.12) |
|  | TCS | 90.44 (44.45) | 40.88 (17.46) | 40.13 (17.76) | 4.48 (0.64) | 277.82 (184.70) |
|  | BPA | 1.96 (0.88) | 1.62 (0.69) | 1.36 (0.76) | 0.52 (0.38) | 5.40 (2.08) |
|  | BPF | 0.18 (0.09) | 0.13 (0.06) | 0.11 (0.07) | ‹LOQ (‹LOQ) | 0.55 (0.20) |
|  | BPZ | ‹LOQ (‹LOQ) | ‹LOQ (‹LOQ) | ‹LOQ (‹LOQ) | ‹LOQ (‹LOQ) | ‹LOQ (‹LOQ) |
|  | BPS | 0.86 (0.31) | 0.61 (0.30) | 0.31 (0.16) | ‹LOQ (‹LOQ) | 4.61 (1.55) |
|  | BPP | ‹LOQ (‹LOQ) | ‹LOQ (‹LOQ) | ‹LOQ (‹LOQ) | ‹LOQ (‹LOQ) | ‹LOQ (‹LOQ) |
|  | DEP | 4.30 (1.24) | 0.30 (0.13) | 0.87 (0.19) | ‹LOQ (‹LOQ) | 32.70 (9.18) |
|  | DETP | 1.95 (0.63) | 0.76 (0.33) | 0.35 (0.24) | ‹LOQ (‹LOQ) | 9.23 (2.34) |
|  | DEDTP | ‹LOQ (‹LOQ) | ‹LOQ (‹LOQ) | ‹LOQ (‹LOQ) | ‹LOQ (‹LOQ) | ‹LOQ (‹LOQ) |
|  | Pollutant | Mean | Geometric mean | Median | Minimum | Maximum |
| ***Blood*** |  |  |  |  |  |  |
|  | Lead | 62.96 | 53.69 | 53.57 | 22.34 | 156.96 |
| ***Serum*** |  |  |  |  |  |  |
|  | HCH ALPHA | ‹LOQ | ‹LOQ | ‹LOQ | ‹LOQ | ‹LOQ |
|  | Hexachloro Benzène | ‹LOQ | ‹LOQ | ‹LOQ | ‹LOQ | 0.09 |
|  | HCH Gamma | ‹LOQ | ‹LOQ | ‹LOQ | ‹LOQ | ‹LOQ |
|  | HCH Beta | ‹LOQ | ‹LOQ | ‹LOQ | ‹LOQ | ‹LOQ |
|  | ALDRINE | ‹LOQ | ‹LOQ | ‹LOQ | ‹LOQ | ‹LOQ |
|  | Oxychlordane | ‹LOQ | ‹LOQ | ‹LOQ | ‹LOQ | ‹LOQ |
|  | t-HEPTEP | ‹LOQ | ‹LOQ | ‹LOQ | ‹LOQ | ‹LOQ |
|  | 2,4’DDE | ‹LOQ | ‹LOQ | ‹LOQ | ‹LOQ | ‹LOQ |
|  | t-CHLORDANE | ‹LOQ | ‹LOQ | ‹LOQ | ‹LOQ | ‹LOQ |
|  | t-NONACHLOR | ‹LOQ | ‹LOQ | ‹LOQ | ‹LOQ | ‹LOQ |
|  | DIELDRINE | ‹LOQ | ‹LOQ | ‹LOQ | ‹LOQ | ‹LOQ |
|  | 2,4’DDT | ‹LOQ | ‹LOQ | ‹LOQ | ‹LOQ | ‹LOQ |
|  | ENDRINE | ‹LOQ | ‹LOQ | ‹LOQ | ‹LOQ | ‹LOQ |
|  | c-NONACHLOR | ‹LOQ | ‹LOQ | ‹LOQ | ‹LOQ | ‹LOQ |
|  | ENDOSULFAN | ‹LOQ | ‹LOQ | ‹LOQ | ‹LOQ | ‹LOQ |
|  | 4,4'DDE | 3.02 | 1.69 | 1.46 | ‹LOQ | 9.20 |
|  | PCB 118 | ‹LOQ | ‹LOQ | ‹LOQ | ‹LOQ | ‹LOQ |
|  | PCB 138 | ‹LOQ | ‹LOQ | ‹LOQ | ‹LOQ | 0.24 |
|  | PCB 153 | 0.09 | 0.08 | 0.08 | ‹LOQ | 0.20 |
|  | PCB 180 | 0.086 | 0.074 | 0.06 |  | 0.31 |
|  | PFOA | 0.49 | 0.47 | 0.48 | 0.25 | 0.85 |
|  | PFOS | 0.58 | ‹LOQ | 0.50 | ‹LOQ | 1.54 |
|  | PFHpA | ‹LOQ | ‹LOQ | ‹LOQ | ‹LOQ | 0.06 |
|  | PFHxS | 0.20 | 0.18 | 0.17 | ‹LOQ | 0.44 |
|  | PFNA | 0.16 | 0.12 | 0.11 | ‹LOQ | 0.36 |
|  | PFDA | ‹LOQ | ‹LOQ | ‹LOQ | ‹LOQ | 0.21 |
|  | PCP | ‹LOQ | ‹LOQ | ‹LOQ | ‹LOQ | 102.4^a^ |
|  | 4-OH CB187 | 7.48^a^ | ‹LOQ | 2.44^a^ | ‹LOQ | 20.4^a^ |
|  | 2,4,6-TBP | ‹LOQ | ‹LOQ | ‹LOQ | ‹LOQ | ‹LOQ |
|  | 4-OH CB 107 | ‹LOQ | ‹LOQ | ‹LOQ | ‹LOQ | ‹LOQ |
|  | 3-OH CB 138 | ‹LOQ | ‹LOQ | ‹LOQ | ‹LOQ | ‹LOQ |
|  | 3-OH CB 146 | ‹LOQ | ‹LOQ | ‹LOQ | ‹LOQ | ‹LOQ |
|  | 3-OH CB 153 | ‹LOQ | ‹LOQ | ‹LOQ | ‹LOQ | ‹LOQ |
|  | 6-OH BDE 47 | ‹LOQ | ‹LOQ | ‹LOQ | ‹LOQ | ‹LOQ |
|  | Pollutant | Mean | Geometric mean | Median | Minimum | Maximum |
|  | 3-OH CB 180 | ‹LOQ | ‹LOQ | ‹LOQ | ‹LOQ | ‹LOQ |
|  | 4-OH CB 172 | ‹LOQ | ‹LOQ | ‹LOQ | ‹LOQ | ‹LOQ |
|  | 5-OH BDE 47 | ‹LOQ | ‹LOQ | ‹LOQ | ‹LOQ | ‹LOQ |
|  | TBBPA | ‹LOQ | ‹LOQ | ‹LOQ | ‹LOQ | ‹LOQ |
|  | 5-OH BDE 99 | ‹LOQ | ‹LOQ | ‹LOQ | ‹LOQ | ‹LOQ |
|  | PBDE47 | ‹LOQ | ‹LOQ | ‹LOQ | ‹LOQ | 15.06^a^ |
|  | PBDE 153 | ‹LOQ | ‹LOQ | ‹LOQ | ‹LOQ | 13.45^a^ |
|  | PBDE 28 | ‹LOQ | ‹LOQ | ‹LOQ | ‹LOQ | 8.63^a^ |
|  | PBDE 100 | ‹LOQ | ‹LOQ | ‹LOQ | ‹LOQ | 4.58^a^ |
|  | PBDE 99 | ‹LOQ | ‹LOQ | ‹LOQ | ‹LOQ | 22.02^a^ |
|  | PBDE 154 | ‹LOQ | ‹LOQ | ‹LOQ | ‹LOQ | ‹LOQ |
|  | PBDE 183 | ‹LOQ | ‹LOQ | ‹LOQ | ‹LOQ | ‹LOQ |
|  | PBDE 209 | ‹LOQ | ‹LOQ | ‹LOQ | ‹LOQ | ‹LOQ |

^a^ : concentration in pg/mL

Table S5: Mean, geometric mean, median and range concentrations in urine [µg/L (µg/g creatinine)] and at both blood and serum in µg/L or pg/mL.
